# Deep Learning-Enabled Multi-Omics Integration: A New Frontier in Precise Drug Target Discovery

**DOI:** 10.3390/biology15050410

**Published:** 2026-03-02

**Authors:** Yufei Ren, Haotian Bai, Jihan Wang, Yanning Yang, Yangyang Wang

**Affiliations:** 1 School of Physics and Electronic Information, Yan’an University, Yan’an 716000, China; 2512102015@stu.yau.edu.cn (Y.R.); 2512104008@stu.yau.edu.cn (H.B.); 2 Yan’an Medical College, Yan’an University, Yan’an 716000, China; jihanwang@yau.edu.cn

**Keywords:** deep learning, multi-omics integration, drug target discovery, precision medicine

## Abstract

Precise drug target discovery is pivotal to mitigating the escalating costs and high attrition rates that characterize pharmaceutical research and development. Given that traditional single-omics methods often fail to elucidate the systemic complexity of human diseases, deep learning (DL)-enabled multi-omics integration has emerged as a transformative frontier. This review systematically summarizes the advancements in DL-driven multi-omics integration for drug target discovery. First, the multi-omics data foundation and integration strategies are delineated, followed by an exploration of the DL architectures utilized for processing such data. Subsequently, the efficacy of DL-driven multi-omics integration is examined regarding the identification of novel disease drivers, prediction of synthetic lethality interactions, and prioritization of therapeutic targets. Finally, addressing persistent challenges related to data sparsity, model interpretability, and target druggability and validation hurdles, emerging opportunities driven by Generative AI, Large Multimodal Models (LMMs), Explainable AI (XAI), and multidimensional feasibility assessment frameworks are discussed in the context of advancing precision medicine.

## Simple Summary

The identification of novel drug targets is essential for developing effective therapies and reducing the substantial costs and high failure rates in pharmaceutical research. Traditional analytical methods focusing on a single biological layer often fail to capture the systemic complexity of human diseases. This review summarizes advancements in computational frameworks that integrate diverse biological data, including genes, proteins, and metabolites, to facilitate drug target discovery. We examine how these methodologies identify disease drivers, predict genetic interactions, and prioritize potential therapeutic candidates. Furthermore, this work evaluates critical challenges such as data sparsity, the limited interpretability of models, and the necessity of assessing the chemical and clinical feasibility of predicted targets. By addressing data inconsistencies and establishing transparent, comprehensive evaluation frameworks, these integrated approaches offer the potential to advance precision medicine and enhance the delivery of effective treatments to patients.

## 1. Introduction

The transition toward precision medicine represents a fundamental shift in drug discovery, moving beyond symptom-based treatments to target the molecular drivers of disease [1,2,3]. Despite these advancements, the pharmaceutical industry continues to face the challenges of “Eroom’s Law,” where escalating research costs are met with stagnant approval rates [4,5,6]. This trend is largely driven by high attrition in late-stage clinical trials, often resulting from the selection of sub-optimal or non-druggable targets [7,8,9]. Consequently, the identification of high-confidence therapeutic targets has become the pivotal bottleneck in improving clinical success rates. Traditionally, target discovery has relied on reductionist single-omics methods, such as Genome-Wide Association Studies (GWASs) or differential expression analysis [10,11]. However, these approaches frequently fail to capture the systemic complexity of human diseases, overlooking integrated regulatory mechanisms involving epigenetic modifications, post-translational interactions, and metabolic perturbations [12].

To resolve these limitations, the integration of multi-omics data, including genomics, transcriptomics, proteomics, and metabolomics, has become essential for reconstructing the functional molecular networks underlying pathogenesis [13,14,15]. Unlike isolated datasets, multi-omics integration provides complementary biological insights, enabling researchers to reconstruct complex molecular networks underlying disease pathogenesis [16,17,18]. Consequently, multi-omics integration facilitates the identification of therapeutic targets that are biologically actionable and clinically relevant [19,20,21]. However, transforming these high-dimensional data into actionable medical knowledge presents formidable computational challenges. Multi-omics datasets are characterized by high heterogeneity, sparsity, scale differences, and the “curse of dimensionality,” rendering traditional bioinformatics and statistical methods insufficient for effective integration [9,22,23].

**Figure 1:**
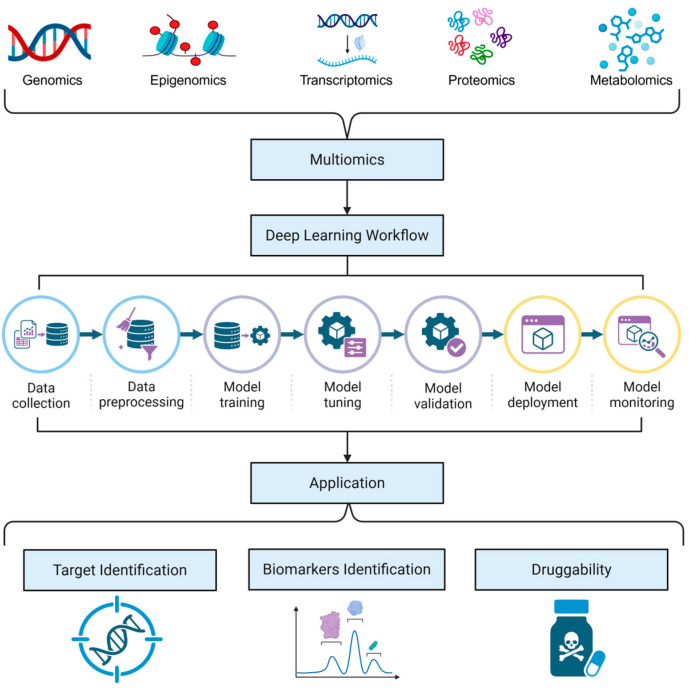
DL-enabled multi-omics integration workflow for therapeutic applications. The central Deep Learning Workflow delineates the comprehensive model development lifecycle, sequentially progressing through data collection, preprocessing, training, tuning, validation, deployment, and monitoring. This systematic pipeline transforms heterogeneous biological data into actionable insights for critical downstream applications, including target identification, biomarker discovery, and druggability assessment.

DL represents a transformative breakthrough in handling this biological complexity, offering capabilities far beyond traditional methods [24,25,26]. While classical algorithms require labor-intensive feature engineering, DL models excel at extracting high-level abstractions from raw data through automated representation learning. Specific architectures have been tailored to overcome the hurdles of multi-omics analysis: variational autoencoders (VAEs) serve to denoise and compress sparse omics data into robust low-dimensional representations [27], convolutional neural networks (CNNs) extract local patterns from sequences or spatial data [28], and graph neural networks (GNNs) incorporate prior knowledge to preserve the structural topology of biological interactions [29]. Furthermore, the transformer architecture employs attention mechanisms to differentially weight omics modalities and capture long-range dependencies within biological sequences [30], as well as diffusion models for generative data augmentation. Recent advancements also include large-scale pre-trained foundation models, which enable accurate predictions in data-scarce scenarios [31,32], effectively overcoming the sample size bottleneck. By addressing the intrinsic complexity of multi-omics data, these DL-driven frameworks transcend traditional limitations to support a holistic characterization of disease signatures. This capability empowers researchers to capture intricate non-linear dependencies and pinpoint high-confidence therapeutic vulnerabilities, thereby catalyzing a shift toward data-driven precision medicine. Figure 1 provides a schematic overview of this DL workflow, illustrating the systematic progression from multi-omics data acquisition through the DL pipeline to downstream therapeutic applications.

This review aims to comprehensively and systematically summarize the application of DL-driven multi-omics integration in drug target discovery. First, we outline the multi-omics data foundation underpinning target discovery, highlighting the biological significance of different omics layers and the key public databases that support data-driven target identification. We then provide a structured overview of multi-omics integration strategies and dimensionality reduction technologies, and we systematically examine the major DL architectures for multi-omics integration tasks. Subsequently, we discuss the applications of DL-enabled multi-omics integration in drug target discovery tasks, including disease driver identification, synthetic lethality prediction, and target prioritization. Finally, we critically examine the current limitations of DL-enabled multi-omics approaches, with a particular focus on data quality, model interpretability, and experimental validation, and discuss emerging opportunities driven by explainable artificial intelligence, generative artificial intelligence, large multimodal models, and multidimensional feasibility assessment frameworks that may shape the future of precision medicine.

## 2. The Multi-Omics Data Foundation for Drug Target Discovery

### 2.1. Types of Multi-Omics and Their Biological Significance

Genomics and epigenomics form the foundational layers of biological regulation. Genomics analyzes static DNA-level information, including single-nucleotide variations (SNVs), copy number variations (CNVs), and structural rearrangements, to gain an in-depth understanding of genetic or somatic mutations that may produce actionable dependencies [33,34,35]. While genomic changes are primary indicators of abnormal signaling pathways [36,37], they are intrinsically regulated by the epigenome. Epigenomics captures DNA methylation patterns, histone modifications, chromatin accessibility, and three-dimensional genome structure [38,39,40]. Unlike genetic mutations, the epigenetic state is reversible, making it a highly promising drug target. Epigenome analysis provides mechanistic insights into transcriptional dysregulation and can reveal epigenetic regulation nodes as potential therapeutic intervention points [41,42,43].

Downstream of these regulatory layers, the functional state of the cell is captured by transcriptomics, proteomics, and metabolomics. Transcriptomics characterizes the regulation of gene expression dynamics and RNA levels [9,44]. By quantifying mRNA abundance, alternative splicing, and non-coding RNA activity, it reflects the functional consequences of genomic perturbations and reveals dysregulated pathways or expression-based biomarkers [45,46,47]. However, gene expression does not always correlate with protein levels, necessitating proteomics to quantify protein abundance, post-translational modifications (PTMs), and protein–protein interactions (PPIs) [48,49,50]. Since most therapeutic drugs bind directly to proteins, proteomics accurately reflects drug-targeted biology, identifying pathway bottlenecks and active signaling modules [51,52,53]. Furthermore, metabolomics investigates small-molecule metabolites that reflect the final biochemical state of cells [54,55]. As metabolic reprogramming is a hallmark of many diseases, this layer can reveal regulatory mechanisms of rate-limiting enzymes and metabolic bottlenecks, providing viable drug targets. Complementing these bulk-level profiles, single-cell omics techniques, including scRNA-seq, scATAC-seq, and spatially resolved multi-omics, introduce cellular resolution into target discovery. These methods can identify cell type-specific targets, rare subpopulations, tumor microenvironment interactions, and heterogeneity-driven drug resistance mechanisms [56,57,58]. These omics layers collectively constitute a network of interconnected molecules. Accurate identification of reliable drug targets is made possible by the integration of these multi-omics data, which offers a systemic biological understanding surpassing that of any single approach. A comprehensive summary of these omics layers, detailing their key features and specific implications for drug target discovery, is provided in Table 1.

### 2.2. Key Databases for Drug Target Discovery

A robust data infrastructure is fundamental for integrating high-dimensional biomedical datasets. Numerous specialized repositories, encompassing genomic variants to protein–ligand affinities and supporting programmatic access via APIs, constitute an essential ecosystem for training AI models (Table 2). The Open Targets Platform serves as a gold standard, integrating multi-source evidence to provide quantified gene–disease association scores. These “ground truth” labels enable supervised deep learning models to characterize druggable targets beyond simple correlations. Additionally, STRING offers curated protein–protein interaction networks, providing the topological foundation for Graph Neural Networks to identify regulatory hubs via network propagation. Together with resources summarized in Table 2, such as GTEx for tissue specificity, DepMap for cancer dependencies, and DrugBank for pharmacological profiles, these databases underpin modern AI-driven target discovery.

## 3. Strategies and DL Architectures Enabling Multi-Omics-Driven Target Discovery

Multi-omics integration provides a holistic view of disease biology but introduces severe dimensionality and heterogeneity challenges. DL addresses these challenges by learning abstract representations from complex biological networks [78], thereby enabling more precise drug target discovery. This section reviews the integration strategies, dimensionality reduction technologies and the principal DL architectures that currently drive drug target discovery based on multi-omics.

### 3.1. Strategies for Multi-Omics Integration

**Figure 2:**
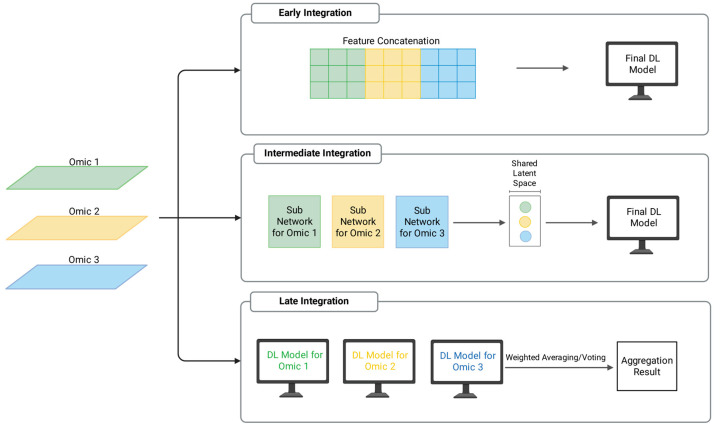
Three paradigms for multi-omics data integration. Early Integration involves concatenating raw features from diverse sources into a unified input matrix prior to model training. Intermediate Integration processes each data type through separate network branches to extract modality-specific representations, which are subsequently merged in a shared latent space to capture cross-modal patterns. Late Integration trains independent models for each modality, and their individual outputs are aggregated at the decision level using methods such as weighted averaging to produce the final prediction.

The strategic selection between single-omics and multi-omics approaches is governed by a fundamental trade-off between statistical robustness and biological depth. Single-omics models remain appropriate for small-sample regimes to mitigate “the curse of dimensionality” and ensure model stability. However, multi-omics integration is necessitated when resolving the synergistic regulatory mechanisms of complex diseases is paramount. In the context of DL-driven multi-omics integration, DL frameworks typically employ three fusion paradigms (Figure 2). Early integration concatenates raw features into a single input vector but often struggles with heterogeneous data distributions and modality-specific noise [79]. Late integration aggregates predictions from independent models [80,81]; while preserving distinct patterns, it typically overlooks critical synergistic interactions. Intermediate integration has become the primary focus in current research [9]. By fusing latent representations from separate network branches, this strategy effectively characterizes non-linear cross-talk [82], facilitating the extraction of robust biological signals as evidenced by frameworks such as MOGONET and OmiEmbed [44,83].

### 3.2. Dimensionality Reduction and Manifold Learning

Given the high-dimensional and sparse nature of multi-omics and molecular dynamics data, dimensionality reduction serves as a critical bridge between raw biological information and interpretable feature learning. Beyond standard visualization techniques like Uniform Manifold Approximation and Projection (UMAP) that preserve local topology [84], deep learning has revolutionized the extraction of latent dynamics from molecular simulations. Notably, approaches such as molecular dynamics-based Markov State Models (MD-MSMs) and VAMPnets utilize time-lagged autoencoders to extract slow-process dynamics from high-dimensional trajectories [85,86]. To ensure embedding fidelity, metrics like embedding error analysis and Wasserstein Distance are increasingly used to quantify topological preservation [87]. These methods collectively distill complex, high-dimensional biological noise into robust low-dimensional manifolds, facilitating the precise identification of cryptic allosteric sites and dynamic therapeutic targets.

### 3.3. DL Architectures

#### 3.3.1. Autoencoders (AEs) and Variational Autoencoders (VAEs)

**Figure 3:**
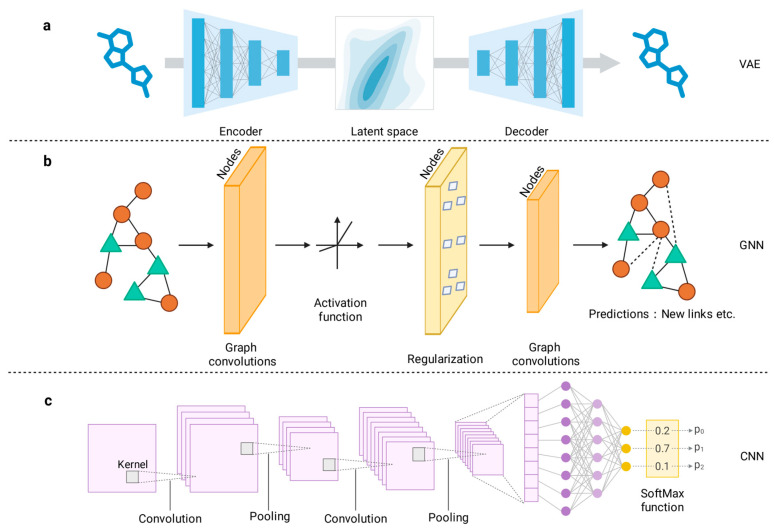
Schematic representation of key deep learning architectures utilized in multi-omics drug discovery. (**a**) Variational Autoencoder (VAE) framework: An encoder network compresses input data (e.g., molecular structures) into a probabilistic latent space, followed by a decoder that reconstructs the original input to learn compressed biological representations. (**b**) Graph Neural Network (GNN) workflow: Biological entities are modeled as nodes within a graph; graph convolution layers aggregate information from neighboring nodes (update step) followed by activation and regularization to predict interactions or properties. (**c**) Convolutional Neural Network (CNN) architecture: Input data (e.g., sequences or feature maps) is processed through successive convolution and pooling layers to extract local invariant patterns, culminating in a fully connected layer for probability-based classification (SoftMax).

Autoencoder (AE)-based frameworks serve as a foundational paradigm in multi-omics integration, effectively reconciling high dimensionality and inherent noise through unsupervised latent representation learning [88,89]. Specialized variants, such as Variational Autoencoders (VAEs), facilitate sophisticated non-linear feature extraction while leveraging generative capabilities to augment limited clinical datasets [90,91]. In multi-modal configurations, modality-specific encoders are typically mapped to a shared latent bottleneck, a mechanism illustrated by the VAE architecture in Figure 3a. This configuration enables cross-omics alignment and synergistic representation learning, thereby providing a robust foundation for downstream therapeutic target discovery. Recent advancements underscore the utility of VAEs and AEs in distilling actionable biological insights from multi-omics noise. A recent study proposed a novel method called FactVAE [92]. The model successfully inferred regulatory peaks for TESK2, a key kinase regulating the actin cytoskeleton. Furthermore, FactVAE identified regulatory elements associated with PHACTR4, a gene known to restrict cancer cell proliferation. Similarly, Pan et al. developed i-Modern [93], an interpretable deep learning framework designed to identify therapeutic targets in glioma. By employing an autoencoder for automated feature extraction, the model integrates heterogeneous multi-omics data, including transcriptomics, miRNA expression, somatic mutations, CNVs, DNA methylation, and proteomics. Notable targets identified included high gene expression of DLL3, specific somatic mutations in IDH1, and high protein expression of IGFBP2, all of which correlated with better prognosis. These findings align with established literature, demonstrating the framework’s efficacy in prioritizing biologically relevant targets.

#### 3.3.2. Graph Neural Networks (GNNs)

Given the inherently relational nature of biological systems, Graph Neural Networks (GNNs) such as Graph Convolutional (GCN) and Graph Attention (GAT) architectures are essential for modeling complex interaction networks. As shown in Figure 3b, these models effectively capture non-Euclidean biological structures by transforming topological relationships into feature embeddings for downstream predictions, including drug target discovery. For instance, Wang et al. developed MOGONET [44], a supervised framework that utilizes GCNs for omics-specific learning and a View Correlation Discovery Network (VCDN) for high-level integration. The model identified critical multi-modal biomarkers for Alzheimer’s Disease (AD). In AD, selected features (e.g., APLN, KIF5A, hsa-miR-423) were biologically validated through GO enrichment analysis linking them to Tau phosphorylation, amyloid-β accumulation, and neuroinflammation. Similarly, Zhang et al. proposed MosGraphFlow [94], a novel integrative graph AI model designed to mine signaling targets from multi-omics data. MosGraphFlow captures multi-level molecular interactions to provide a more comprehensive understanding of AD pathogenesis. By integrating diverse omic layers into a cohesive flow-based graph architecture, the model effectively identifies not only potential biomarkers but also critical signaling pathways that could serve as therapeutic targets. In addition, Niu et al. introduced GLIMS [95], a two-stage gradual-learning framework for cancer gene prediction. The method first employs a semi-supervised hierarchical graph neural network to integrate multi-omics data with protein–protein interaction (PPI) networks for initial candidate identification. It then refines these predictions using an unsupervised approach that incorporates co-splicing networks, effectively capturing critical post-transcriptional regulatory mechanisms to outperform state-of-the-art methods. In a parallel study leveraging GCNs to decipher complex biological interactions, Dai et al. developed DriverOmicsNet [96], a Graph Convolutional Network (GCN) framework that integrates multi-omics data with protein–protein interaction (PPI) networks and weighted gene correlation network analysis (WGCNA). The model demonstrated robust predictive performance by identifying key hub genes, such as ANK2 in stomach adenocarcinoma (STAD) and ACTB in skin cutaneous melanoma (SKCM), which are associated with immune checkpoint response and immune cell infiltration, respectively. Moreover, Li et al. developed CGMega [97], an explainable deep learning framework based on graph attention networks. In acute myeloid leukemia (AML), the framework identified 396 candidate genes and revealed patient-specific gene modules, providing high-order mechanistic insights into cancer heterogeneity and development. Ultimately, by explicitly modeling biological interactions, GNNs offer a distinct capability to uncover network-based therapeutic vulnerabilities that conventional flat-data approaches might overlook.

#### 3.3.3. Convolutional Neural Networks (CNNs)

Although traditionally established in image processing, Convolutional Neural Networks (CNNs) have been effectively adapted to extract features from omics data [98]. Figure 3c demonstrates the standard workflow of this architecture. 1D-CNNs are frequently applied to genomic sequence analysis, such as predicting transcription factor binding sites, or for extracting latent features from chemical structures represented as SMILES strings [99,100], whereas 2D-CNNs are essential for capturing spatial patterns in spatial omics or transforming heterogeneous tabular data into structured feature maps [101]. Demonstrating the utility of 1D-CNNs for quantitative omics, Zompola et al. developed Omics-CNN to analyze complex biological datasets [100]. Applied to the study of ischemic stroke, the pipeline revealed a biosignature associated with the sialic acid metabolism pathway, identifying it as a novel mechanism and a potential therapeutic target for atherosclerosis-related diseases. Crucially, the model prioritized KRT15, VPRBP, TNFRSF4, and GORASP2 as the most significant contributing transcripts. While confirming the known genetic association of TNFRSF4, the study specifically highlighted GORASP2 as a potential therapeutic target for ischemic injury within the Golgi apparatus, showcasing the capability of deep learning to uncover novel druggable candidates beyond established knowledge. Regarding 2D-CNNs, Alok Sharma et al. proposed the DeepInsight-3D architecture [102]. This method relies on a structured data-to-image conversion approach, thereby enabling the utilization of Convolutional Neural Networks (CNNs). The model effectively integrates different types of omics data and demonstrates high efficacy in discovering underlying significant genes. Furthermore, the integration of CNNs with prior biological knowledge has demonstrated substantial potential in neurodegenerative disease research. Wang et al. developed a deep joint learning diagnostic model for Alzheimer’s disease (AD) by introducing a novel multimodal fusion feature termed “MRI-p value” [103]. In this framework, 3D fusion images are constructed by incorporating genetic *p*-values as a priori knowledge into magnetic resonance imaging (MRI) data. The architecture utilizes a dual-branch CNN, in which one branch employs a Residual Network (ResNet) to extract local pathological features, while the other utilizes attention-based convolutions to capture discriminative spatial patterns across diverse brain regions. This model achieved high diagnostic accuracy across AD, mild cognitive impairment (MCI), and healthy controls. Crucially, it identified six novel genetic targets, such as NTM, MAML2, and PCSK5, thereby demonstrating the efficacy of CNNs in translating complex multimodal omics and imaging data into actionable therapeutic insights. In summary, whether applied to sequence data or spatial constructs, CNNs provide a powerful means to capture local dependencies and structural patterns critical for precise target localization.

#### 3.3.4. Transformer and Attention Mechanism

**Figure 4:**
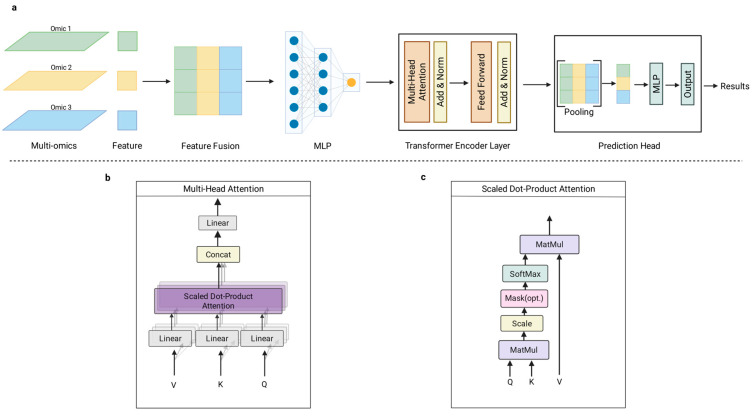
Architecture of a Transformer-based framework for multi-omics data integration and prediction. (**a**) Overall Workflow: The pipeline integrates heterogeneous input data (Multi-omics) through a Feature Fusion layer. The fused representations are processed by a Multi-Layer Perceptron (MLP) before entering the Transformer Encoder Layer, which utilizes attention mechanisms and residual connections (Add & Norm) to extract high-level biological features. The Prediction Head then applies pooling and an MLP to generate the final Results (e.g., drug target identification or biomarker identification). (**b**) Multi-Head Attention: This module enables the model to jointly attend to information from different representation subspaces. Input features are projected into Value (V), Key (K), and Query (Q) vectors, processed in parallel heads, concatenated (Concat), and linearly transformed. (**c**) Scaled Dot-Product Attention: A detailed view of the calculation within each attention head. The mechanism computes the compatibility between queries and keys via matrix multiplication (MatMul), applies scaling and optional masking (Mask), and utilizes a SoftMax function to determine attention weights applied to the values.

The Transformer architecture has emerged as a premier framework for sequence modeling and multi-omics integration. As illustrated in Figure 4, this architecture leverages multi-head attention to orchestrate the fusion of disparate omic layers, capturing intricate non-linear dependencies while transcending the proximity constraints of traditional models [30]. Within biological contexts, Transformer-based frameworks such as DNABERT excel at deciphering the semantic “language” of genomic and proteomic sequences [104,105]. Moreover, a study employed a transformer-based framework to integrate multi-omics data for drug target discovery. For instance, DeePathNet is a transformer-based deep learning model that integrates multi-omics cancer data with biological pathway information to predict drug response and support pathway-level biomarker discovery [106]. By combining genomic, transcriptomic, and other omics inputs within a transformer architecture that encodes pathway interactions, DeePathNet outperforms traditional models in predicting drug responses and identifying biologically relevant features that may serve as candidate therapeutic targets, demonstrating the utility of transformer-driven multi-omics integration for drug target discovery. Researchers developed Precious1GPT [107], a multimodal transformer-based framework that leverages transfer learning to integrate transcriptomic and methylation data along with metadata. The model utilizes feature importance analysis to identify dual-purpose therapeutic targets potentially implicated in both aging processes and age-associated diseases, demonstrating the versatility of transformers in multi-omics representation learning. Taken together, as the dominant architecture in sequence modeling, transformer-based approaches are poised to revolutionize how we interpret the “language” of multi-omics, offering unprecedented interpretability for future drug target discovery pipelines.

#### 3.3.5. Diffusion Models

Diffusion models serve as a powerful generative framework for multi-omics integration, enabling the joint modeling of heterogeneous modalities and the reconstruction of high-fidelity data from sparse, noisy biological landscapes. By capturing complex high-dimensional distributions, these architectures facilitate robust data imputation and conditional generation. For instance, Janson et al. developed idpGAN to directly generate realistic protein conformational ensembles [108], bypassing the need for computationally expensive physics-based iterative sampling. By learning from molecular mechanics simulations, this model produces physically plausible and energetically favorable conformations for unseen proteins, thereby facilitating granular assessments of target druggability. This rapid synthesis allows researchers to account for protein flexibility and dynamic binding sites, a capability essential for identifying non-obvious therapeutic targets that traditional static structural models often overlook. In another distinct application, Luo et al. introduced scDiffusion [109], a conditional generative framework leveraging Latent Diffusion Models (LDMs) to synthesize high-fidelity single-cell data. This model demonstrates exceptional stability in learning complex distributions, enabling the generation of realistic gene expression profiles even for rare cell types with limited training samples. Notably, by employing a unique gradient interpolation strategy, scDiffusion can simulate out-of-distribution data and intermediate cell states between known phenotypes. Extending this generative paradigm to the spatial dimension, diffusion architectures have also been adapted to address the critical challenge of data sparsity in spatially resolved omics. For instance, Li et al. developed stDiff [110], a conditional diffusion framework that leverages single-cell transcriptomics to impute missing signals in spatial transcriptomics (ST) data. Unlike methods relying on simple cell-to-cell similarity, stDiff models the intrinsic correlations of gene expression abundance through a generative denoising process. This approach successfully preserved cellular topological structures and accurately reconstructed complex spatial patterns across diverse datasets, thereby enabling the precise delineation of tissue boundaries and cell populations. These considerations are crucial for translating computational predictions into clinically actionable insights. These advancements collectively provide a robust computational foundation for predicting high-confidence drug targets.

#### 3.3.6. Comparative Summary of DL Architectures for Multi-Omics-Based Target Discovery

The selection of an optimal deep learning framework is not a one-size-fits-all endeavor but rather a strategic decision that depends on the specific biological hypothesis and data modality under investigation. Each architecture discussed above presents distinct trade-offs in terms of computational efficiency, feature extraction capability, and structural compatibility. To provide a consolidated perspective on model selection, Table 3 presents a comparative analysis of these principal deep learning architectures. This summary outlines their core strengths, inherent limitations, and context-specific data applicability, with particular emphasis on two essential dimensions: scalability to large-scale datasets and the potential for mechanistic interpretability. These considerations are crucial for translating computational predictions into clinically actionable insights.

## 4. Deep Learning-Enabled Multi-Omics Integration for Drug Target Discovery

The integration of multi-omics via DL has been successfully applied across multiple stages of the modern target discovery pipeline, demonstrating significant practical application value. This section focuses on its specific utility in identification of novel disease drivers, synthetic lethality prediction, target prioritization.

### 4.1. Identification of Novel Disease Drivers

DL has revolutionized driver identification by prioritizing network-based representation learning over frequency statistics. Graph Neural Networks (GNNs) integrate multi-omics data into the interactome’s topology, identifying “network hubs” that exert systemic influence despite low mutation burdens, thus distinguishing true drivers from passengers [111,112]. Complementing this, Transformers and Variational Autoencoders (VAEs) extend discovery to non-coding and regulatory regions [113,114]. By leveraging self-attention mechanisms to decode long-range sequence dependencies and analyzing latent reconstruction errors, these architectures effectively pinpoint dysregulated elements, uncovering functional drivers that remain invisible to traditional single-omics analyses. Recent studies have demonstrated their ability to pinpoint high-confidence drivers in complex multi-omics landscapes. Ma et al. introduced DeepMAPS [115], a framework utilizing a heterogeneous graph transformer to infer biological networks from single-cell multi-omics data (e.g., CITE-seq and scRNA-ATAC-seq). By jointly embedding cells and genes within a unified graph, the model modeled interpretable cell–gene relations, enabling the precise identification of 13 distinct cell types in lung tumor environments. Crucially, it dynamically integrated chromatin accessibility with gene expression to uncover specific transcription factors (TFs) driving the development states of diffuse large B-cell lymphoma (DSLL), validating these regulatory drivers as potential immuno-therapeutic targets. In parallel, Yang et al. developed Trans-Driver [116], a deep learning model featuring a novel transformer architecture enhanced with kernel-based multi-head self-attention and Dynamic Tanh (DyT) normalization. This design allowed the model to robustly integrate heterogeneous multi-omics features, capturing subtle non-linear associations often missed by standard methods. Applied to TCGA datasets, Trans-Driver identified 269 candidate driver genes with a remarkable 49.1% match rate against the gold-standard Cancer Gene Census (CGC), proving that integrating multi-omics data via advanced attention mechanisms significantly outperforms methods relying solely on somatic mutations. More recently, Huang et al. proposed MOGOLA [117], a supervised multi-omics integration framework that synergizes Graph Convolutional Networks (GCNs) and Graph Attention Networks (GATs) with an Omics-Linked Attention mechanism to optimize feature representation. Crucially, in the analysis of BRCA, the identified biomarkers demonstrated strong alignment with clinically significant pathways and actionable drug targets, thereby reinforcing the rationale for subtype-specific interventions, including HER2-directed therapies. Specifically, the model highlighted dysregulated proliferation characterized by enrichment in the cell cycle and PI3K–Akt signaling pathways. Furthermore, PPI analysis pinpointed key hub genes, including CCNA2, CDK1, and ESR1, which align with established cancer hallmarks and known drug targets. These results corroborate that MOGOLA effectively captures underlying disease-driving mechanisms rather than mere statistical correlations.

Expanding beyond oncology, Dong et al. developed Omicsformer [118], a deep learning framework that integrates transcriptomic, proteomic, and metabolomic data with routine blood analysis for chronic disease risk prediction. The model successfully identified critical molecular drivers across diverse pathological conditions. Specifically, it highlighted HEXIM in cardiac hypertrophy, TG and PC metabolites in cardiovascular and liver diseases, and FOXO3 in Alzheimer’s disease (AD) pathogenesis. These findings validate the model’s capacity to uncover molecular targets regulating oxidative stress, apoptosis, and metabolic reprogramming, underscoring the value of multi-omics integration in systemic risk stratification. Focusing on neurodegenerative pathology, Xie et al. proposed TransFuse [119], a deep fusion model that mimics the dynamic information flow from DNA to RNA and proteins to unravel the molecular mechanisms of AD. By reconstructing interpretable multi-omic sub-networks, the model identified cohesive functional modules that link genetic risk factors to downstream pathology. Notably, it mapped the interaction between the primary AD risk factor APOE and the transcription factor EGR1, while simultaneously capturing the MAPT gene and tau_PHF1_S404 peptide, confirming the critical role of tau phosphorylation in neurofibrillary tangle formation. Beyond established markers, TransFuse elucidated a complex inflammatory axis involving APP, CD44, and EGR1, and highlighted the functional connectivity between the ANGPT2 gene and hub peptide PIK3R1, implicating neuroinflammation and blood–brain barrier integrity in disease progression. These findings were further validated through eQTL analysis, which confirmed that the identified Single-Nucleotide Polymorphisms (SNPs) exert tissue-specific effects in the frontal cortex. Moreover, pathway enrichment revealed significant crosstalk between VEGF and EPH signaling, suggesting that the dysregulation of angiogenesis and synaptic maintenance pathways acts as a fundamental driver of AD pathology. Addressing cardiovascular health, Luo et al. developed CardiOmicScore [120], a multitask deep learning framework for personalized risk assessment of six common cardiovascular diseases (CVDs). Using UK Biobank data, the model utilizes MetNet and ProNet to profile 168 metabolites and 2920 proteins, capturing complex non-linear interactions often missed by traditional models. The resulting MetScore and ProScore are robust predictors that enhance risk stratification up to 15 years prior to disease onset when integrated with clinical factors. Furthermore, the framework identifies critical CVD-related molecular pathways and biomarkers for conditions like heart failure and stroke, demonstrating the capacity of multitask learning to uncover targets for primary prevention and precision medicine. Collectively, These findings demonstrate that by decoding complex, non-linear interactions within multi-omics data, deep learning frameworks can effectively illuminate functional drivers that remain invisible to traditional single-omics analyses.

### 4.2. Synthetic Lethality Prediction for “Undruggable” Targets

Synthetic lethality (SL) offers a pivotal strategy for targeting historically “undruggable” malignancies by exploiting genotype-specific survival vulnerabilities. By inhibiting synergistic lethal partners, SL induces cell death exclusively in mutant contexts while preserving healthy tissue. DL has fundamentally reshaped this landscape, transitioning from labor-intensive screenings to data-driven computational inference. Unlike traditional methods limited to linear associations, DL frameworks integrate heterogeneous multi-omics data—spanning mutations and transcriptomics—to reconstruct complex genetic dependencies. Central to this innovation are architectures like Graph Neural Networks (GNNs), which map the global topology of molecular interactions. By propagating biological signals across multi-modal networks, these models unveil latent functional connections invisible to reductionist approaches, thereby accelerating the discovery of robust targets for precision oncology [121,122]. Recently, several studies have explored this direction. For example, Fan et al. proposed MLEC-iSL [123], a framework that introduces the concept of “SL connectivity” as an intermediate learning objective. By incorporating a Graph Transformer to capture long-range dependencies, the model bridged the gap between computation and wet-lab validation: a purposely designed CRISPR-Cas9 double-knockout (CDKO) experiment guided by the model achieved a confirmed synthetic lethality rate of 46.8% among the predicted candidates, vastly outperforming the 7.2% success rate of unguided screens. This high concordance confirms that modeling the global connectivity of genetic networks can effectively pinpoint functional partners for specific genetic backgrounds. Similarly, Lee and Nam developed KG-Slomics [124], a relational graph attention network that addresses the challenge of generalization across different cancer types by integrating an extensively updated knowledge graph with cell line-specific multiomics data. By embedding topological information from biological networks alongside genomic features such as gene expression and mutations, the model dynamically captures context-dependent interactions that static networks often miss. KG-SLomics demonstrated superior predictive performance over existing baselines and successfully identified novel therapeutic targets, such as the synthetic lethal relationship between TP53 and PDGFRB, which was further corroborated through patient survival analysis and drug response validation. Furthermore, Fan et al. introduced MVGCN-iSL [125], a multi-view graph convolutional network integrating five biological graph features and multi-omics data to predict cell-specific SL. The model employs max pooling and a deep neural network (DNN) for final prediction. Researchers validated MVGCN-iSL on the K562 cell line (100,128 samples; 1523 SL pairs) and the Jurkat cell line (74,691 samples; 373 SL pairs). Results demonstrated strong predictive performance and robust generalization to novel genes, confirming the effectiveness of integrating multiple graph features and multi-omics data for SL prediction. Addressing the need for context-aware predictions, Pu et al. proposed SLWise [126], a deep learning framework designed to identify cell-line specific SL. By incorporating a self-attention module to integrate cell-specific multi-omics data into graph representations, the model captures dynamic gene relationships. In the A375 cell line, SLWise predicted a novel SL interaction between BCL2L2 and WEE1. Further investigation revealed that the knockdown of these genes led to abnormalities in key drivers CNOT9 and RHOA. Notably, given RHOA’s established function in driving tumorigenesis and metastatic dissemination across diverse malignancies, it is widely recognized as a promising candidate for therapeutic intervention. These findings demonstrate the model’s capacity to uncover mechanisms underlying cell-specific therapeutic vulnerabilities. Collectively, these advancements underscore the capability of deep learning to decode complex, context-dependent genetic dependencies, thereby unlocking new avenues for precision therapy in cancers traditionally deemed undruggable.

### 4.3. Drug Target Prioritization

Identifying putative therapeutic entities is merely the prelude; the definitive challenge lies in prioritizing candidates to select those with the highest clinical probability. Target prioritization represents a sophisticated multi-criteria decision-making process that DL transforms into a “learning-to-rank” task. Functioning as evidence fusion engines, DL frameworks integrate heterogeneous data streams, encompassing high-dimensional multi-omics features, ranging from transcriptomic variations to proteomic stability, alongside complex network topologies. By employing strategies such as adaptive graph learning and attention mechanisms, these models automatically weigh the reliability of diverse evidence chains. This process effectively filters noisy correlations and quantifies the systemic value of candidates, generating a robust, convergent hierarchy of actionable targets optimized for experimental validation. A recent study by Tripathy et al. proposed GNNRAI [127], an explainable graph neural network framework that integrates multi-omics data with prior knowledge to prioritize Alzheimer’s disease (AD) biomarkers. By calculating an integrated Target Risk Score (TRS), the model successfully identified APP, APOE, LGMN, and LTF as top-ranked candidates, all of which fell within the top 2% of scored genes. Crucially, these rankings align with established pathology: APP and APOE are canonical drivers of amyloid-β (Aβ) and tau aggregation, while LTF serves as a predictor of Aβ burden. Furthermore, the identification of LGMN (δ-secretase) highlights the model’s capacity to pinpoint critical enzymes involved in the pathogenic cleavage of both tau and APP, validating its effectiveness in capturing key disease biodomains. Advancing the interpretability of feature prioritization, Elmarakeby et al. developed P-NET [128], a biologically informed deep learning architecture designed to unify diverse molecular data types, including somatic mutations, copy number variations, and gene fusions, within a single predictive framework. Unlike traditional statistical methods that process features in isolation, P-NET assigns differential weights to these inputs based on their predictive utility. The model innovatively embeds hierarchical prior biological knowledge directly into the neural network structure, creating an interpretable computational graph that facilitates both clinical prediction and biological discovery. By visualizing the model’s internal architecture, researchers can decipher the multi-level biological pathways driving disease progression. In the context of castration-resistant prostate cancer (CRPC), P-NET not only corroborated established drivers such as AR, PTEN, TP53, and RB1 but also prioritized MDM4 as a critical therapeutic target. This discovery was experimentally validated, suggesting that MDM4 inhibitors could serve as an effective precision therapy for metastatic patients harboring wild-type TP53. Similarly, Yang et al. developed PI4AD [129], a computational medicine framework designed to prioritize therapeutic targets for AD by bridging the gap between genetic associations and pathway-level biology. PI4AD successfully recovered clinically validated targets within the top 1% of prioritized genes, assigning high rankings to APP (18th), ESR1 (61st), KIT (95th) and PDGFRB (100th), which are targets of drugs in Phase III trials or approved clinical use. Furthermore, the framework utilized artificial neural networks to construct self-organizing prioritization maps that distinguish AD-specific molecular signatures, characterized by cell motility and neurotrophin signaling, from those of comorbid neuropsychiatric disorders. The study further identified a 51-gene pathway crosstalk network, revealing Ras signaling as a central therapeutic hub; notably, the combinatorial removal of Ras-pathway nodes (HRAS, KRAS, NRAS) and BCL2 was shown to disrupt 49.0% of the network’s connectivity, suggesting a novel strategy to target neuroinflammation and synaptic dysfunction beyond traditional amyloid/tau paradigms. To address the complexity of target identification in neurodegenerative disorders, Tsuji et al. developed a computational framework utilizing a deep autoencoder to prioritize putative target genes for AD [130]. By leveraging the non-linear feature extraction capabilities of deep learning, the study successfully identified key pathogenicity-associated genes, including DLG4, EGFR, RAC1, SYK, PTK2B, and SOCS1. Furthermore, the framework bridged the gap between target discovery and therapeutic intervention by inferring promising candidates for drug repurposing based on these prioritized targets. Notably, the model identified compounds such as tamoxifen, bosutinib, and dasatinib as potential therapeutics for AD. Taken together, these studies exemplify the capacity of deep learning to bridge the gap between high-throughput multi-omics data and the identification of high-confidence, translatable therapeutic targets.

### 4.4. Summary of Representative Studies

This section presents a comprehensive summary of representative studies that have successfully leveraged DL-enabled multi-omics integration for drug target discovery. Table 4 systematically categorizes these pivotal works, detailing the specific Study/Model, the utilized Datasets, and the underlying DL Architectures (e.g., GNNs, Transformers, VAEs). Crucially, we highlight the Key Discovery & Validation for each entry, demonstrating how these computational frameworks translate complex multi-omics data into actionable biological insights. These studies collectively address critical challenges in the field, encompassing the identification of novel disease drivers, synthetic lethality prediction, and the robust prioritization of therapeutic targets.

## 5. Challenges and Future Perspectives

Despite the transformative potential of DL in target discovery, the progression from in silico validation to clinical utility remains obstructed by fundamental structural barriers. To dismantle these impediments, a paradigm shift is required—one that moves beyond standard algorithms to address the intrinsic limitations of data sparsity and heterogeneity, resolves the crisis of model opacity, and acknowledges that even biologically relevant targets may lack pharmacological tractability, necessitating a holistic evaluation across biological, chemical, and clinical dimensions.

### 5.1. Data Sparsity and Heterogeneity

Although deep learning has demonstrated strong capability in modeling complex biological systems, its effectiveness in multi-omics integration is fundamentally constrained by the intrinsic sparsity and heterogeneity of high-dimensional data. Consequently, even sophisticated algorithms struggle to extract robust biological signals from such sparse, high-dimensional landscapes, where data incompleteness inevitably degrades downstream predictive accuracy. To address these specific bottlenecks, the field is pivoting toward Generative AI and Large Multi-Modal Models (LMMs) [138,139]. Rather than relying solely on direct feature aggregation, these generative frameworks tackle data sparsity by augmenting limited datasets while simultaneously overcoming heterogeneity through harmonizing disparate omics features into a unified latent space, thereby enabling more stable, generalizable, and robust predictions in data-scarce and highly heterogeneous scenarios.

### 5.2. The Interpretability Crisis

Compounding the challenge is the inherent opacity of DL models. High predictive accuracy remains insufficient without mechanistic transparency; the lack of robust Explainable AI (XAI) frameworks to distinguish causality from correlation sustains a significant trust gap between computational predictions and biological reality. This opacity compromises clinical trust, as the inability to provide biological justification for predictions creates a barrier to their integration into real-world therapeutic decision-making. To bridge this divide, the field must pivot from correlation-based learning toward causality-aware XAI frameworks [140,141]. This evolution transforms models from opaque “pattern matchers” into “causal inference engines,” enabling the simulation of in silico perturbations to distinguish biologically viable intervention points from mere statistical artifacts.

### 5.3. Target Druggability and Validation Hurdles

While deep learning models excel at identifying biologically relevant targets, biological significance does not equate to pharmacological tractability. A predicted target may lack a druggable binding pocket, exhibit unfavorable pharmacokinetic properties, or pose safety liabilities due to structural homology [142]. Consequently, the transition from computational nomination to therapeutically viable intervention remains a critical bottleneck. Furthermore, even high-confidence candidates require resource-intensive wet-lab validation where attrition rates remain substantial despite promising in silico metrics [143]. To bridge these gaps, the field must shift toward a holistic paradigm that evaluates candidates within a multidimensional landscape of biological, chemical, and clinical feasibility, thereby enhancing the translational success of AI-driven discovery.

## 6. Conclusions

In this review, we systematically summarize the major applications of deep learning-driven multi-omics integration in the field of drug target discovery. Deep learning-enabled multi-omics integration represents a highly promising approach for accelerating precise drug target discovery. We present a complete analytical workflow, beginning with an overview of the foundations of multi-omics data and a systematic examination of integration strategies—spanning early, intermediate, and late integration. Furthermore, we discuss the pivotal role of dimensionality reduction and manifold learning in extracting latent dynamics and interpretable features from high-dimensional datasets. This is followed by a detailed discussion of advanced DL architectures, including autoencoders, graph neural networks, convolutional neural networks, and Transformer-based models, and their respective roles in analyzing heterogeneous multi-omics data. Importantly, our analysis emphasizes the practical utility of DL in addressing key tasks in drug target discovery, particularly in the identification of disease-driving factors, the prediction of synthetic lethality, and the prioritization of therapeutic targets.

Despite these advances, structural barriers regarding data sparsity, heterogeneity, model opacity, and challenges in druggability and experimental validation persist. Future progress hinges on the advancement of Generative AI and LMMs to mitigate data scarcity and harmonize heterogeneity, causal XAI to decipher mechanistic logic, and the development of holistic, multidimensional frameworks that integrate biological, chemical, and clinical insights to systematically evaluate the pharmacological feasibility of identified targets. Surmounting these challenges is essential to reconcile in silico predictions with clinical reality, ultimately catalyzing the development of next-generation precision therapeutics.

## Figures and Tables

**Table 1:** Summary of Multi-Omics Layers, Key Features, and Implications for Drug Target Discovery.

Omics Layer	Key Features Analyzed	Biological Role	Relevance to Target Discovery	Refs
Genomics	SNVs, CNVs, structural rearrangements	Foundational static DNA information; indicators of abnormal signaling	Identifies genetic/somatic mutations creating actionable dependencies	[59,60]
Epigenomics	DNA methylation, histone modifications, chromatin accessibility, 3D structure	Dynamic regulation of gene expression; intrinsically regulates genomics	Reversible states offer promising therapeutic points; reveals nodes of transcriptional dysregulation.	[61]
Transcriptomics	mRNA abundance, alternative splicing, non-coding RNA	Characterizes gene expression dynamics and RNA regulation	Reflects functional consequences of perturbations; uncovers expression-based biomarkers.	[62]
Proteomics	Protein abundance, PTMs, PPls	Quantifies functional executors; correlates with drug binding sites	Reflects drug-targeted biology directly; identifies pathway bottlenecks and signaling modules.	[63,64]
Metabolomics	Small-molecule metabolites	Represents the final biochemical state and metabolic phenotype	Reveals rate-limiting enzymes and metabolic bottlenecks as viable targets.	[65]
Single-cell Omics	scRNA-seq, scATAC-seq, spatial multi-omics	Introduces cellular resolution to bulk profiles	Identifies cell-type-specific targets, rare subpopulations, and heterogeneity-driven resistance.	[66,67]

**Table 2:** Key databases in the field of drug target discovery.

Dataset	Description	Link	APIs	Refs
Open Targets	Integrates genetics, somatic mutations, animal models, and drug data to provide scored target–disease associations for prioritization.	https://www.targetvalidation.org/	Yes	[68]
TTD	A comprehensive resource providing information on approved, clinical trial, and failed therapeutic targets with druggability data.	https://idrblab.org/ttd/	Yes	[69]
NHGRI-EBI GWAS Catalog	The most extensive collection of identified genetic variants associated with human traits, essential for causal target identification.	https://www.ebi.ac.uk/gwas	Yes	[70]
BindingDB	a public database of measured small molecule–protein interactions, used for drug development, AI training, and computational methods.	https://www.bindingdb.org/	Yes	[71]
GTEx	Provides genotype data and gene expression profiles (including single-nucleus RNA-seq) across multiple non-diseased human tissues.	https://gtexportal.org/	Yes	[72]
DepMap	Contains genome-wide CRISPR and RNAi perturbation screens to identify essential genes and cancer dependencies (synthetic lethality).	https://depmap.org/portal/	Yes	[73]
KEGG	A pathway-centered resource mapping molecular interactions and reaction networks for target contextualization and orthology-based annotation.	https://www.kegg.jp	Yes	[74]
STRING	A database of known and predicted protein–protein interactions (PPIs), covering both physical associations and functional links.	https://string-db.org/	Yes	[75]
ChEMBL	A manually curated database of bioactive molecules with drug-like properties, providing binding affinity data (Ki, IC50, Kd).	https://www.ebi.ac.uk/chembl/	Yes	[76]
DrugBank	A comprehensive database of drug structures, mechanisms, and target sequences, providing extensive annotated drug–target interactions (DTIs) and pharmacological profiles.	https://go.drugbank.com	Yes	[77]

APIs: Indicates whether the database provides an Application Programming Interface for programmatic data access and integration (“Yes” denotes availability). TTD, Therapeutic Target Database; NHGRI-EBI GWAS Catalog, National Human Genome Research Institute-European Bioinformatics Institute Genome-Wide Association Studies Catalog; GTEx, Genotype–Tissue Expression; DepMap, Cancer Dependency Map; KEGG, Kyoto Encyclopedia of Genes and Genomes; STRING, Search Tool for the Retrieval of Interacting Genes/Proteins.

**Table 3:** Comparative summary of DL architectures for multi-omics-based target discovery.

Architecture Type	Key Strengths	Limitations & Trade-Offs	Data Requirement Preference	Scalability & Interpretability
Autoencoders (AE/VAE)	Unsupervised reduction and denoising; compresses sparse, high-dimensional data into robust latent features.	Prone to overfitting on small datasets; highly sensitive to pre-processing (e.g., normalization); generative outputs require rigorous validation.	Strictly matched multi-omics samples; requires rigorous normalization for latent space alignment.	Scalability: Scales well with large feature matrices. Interpretability: The “black-box” latent space is abstract; post hoc analysis is needed to trace biological meaning.
Graph Neural Networks (GNNs)	Captures relational topology and non-Euclidean interactions; integrates prior biological knowledge.	Computational cost grows exponentially with graph density; susceptible to “oversmoothing” in deep layers, leading to loss of node distinctiveness.	Multi-omics matrices (e.g., RNA-seq, methylation, proteomics) integrated with prior knowledge graphs (PPIs, pathways, etc.).	Scalability: Training is challenging for massive graphs without sampling. Interpretability: GATs allow tracing of edge importance via attention weights.
Transformer & Attention Mechanism	Models long-range dependencies; self-attention mechanisms dynamically weigh feature importance regardless of distance; highly parallelizable.	Data-hungry; requires massive datasets for pre-training to avoid overfitting; quadratic computational complexity with respect to sequence length.	Sequential biological data (DNA/protein), tokenized multi-omics profiles (e.g., scRNA-seq treated as gene sequences), and heterogeneous multi-modal paired data.	Scalability: Computational load is heavy for long sequences. Interpretability: Feature contributions are derived via attention mechanisms.
CNNs	Efficient local feature extraction; excels at spatial pattern recognition in grid-structured data.	Inflexible for unstructured data; struggles with non-Euclidean inputs.	Requires features with local spatial correlations (e.g., genomic sequences or pixel-aligned histopathology-omics images).	Scalability: Very efficient training and inference on GPUs. Interpretability: Saliency maps can highlight regions, but feature logic is often opaque.
Diffusion Models	High-fidelity data generation; capable of data augmentation and modeling complex data distributions (e.g., protein conformations).	Training instability; Diffusion models suffer from slow sampling speeds; risk of “hallucinating” biological artifacts.	Extensive high-fidelity datasets; requires conditional cues (e.g., disease states) for guided synthesis and target discovery.	Scalability: Heavy Computational Burden. Interpretability: Opaque Generative Logic.

**Table 4:** Summary of representative studies utilizing DL-enabled multi-omics integration for drug target discovery.

Study/Model	Dataset	DL Architecture	Key Discovery & Validation	References
MOGONET	Datasets from ROSMAP dataset and TCGA	GCN	It identified top 30 multi-modal biomarkers (mRNA, DNA methylation, miRNA) for Alzheimer’s Disease (AD) and Breast Cancer (BRCA) classification. In AD, selected features (e.g., APLN, KIF5A, hsa-miR-423) were biologically validated through GO enrichment analysis linking them to Tau phosphorylation, amyloid-β accumulation, and neuroinflammation. For BRCA, the model pinpointed subtype-specific markers (e.g., SOX11, FABP7, miRNA-205), which were confirmed by literature to drive tumor proliferation, metabolic reprogramming, and metastasis.	[44]
DeepMAPS	Datasets from GEO, Figshare, 10× Genomics and CNGB	GNN and Transformer	TFs and genes identified in this study serve as potential markers for validation and potential immunotherapeutic targets for DSLL treatment.	[115]
Trans-Driver	Datasets from TCGA and the COSMIC CGC	Transformer	Among approximately 20,000 protein-coding genes, Trans-Driver reported 269 candidate driver genes, of which 132 genes (about 49.1%) were included in the gold standard CGC dataset. Feature contribution analysis further demonstrated that integrating multi-omics data improved performance compared to using somatic mutation data alone.	[116]
MOGOLA	Datasets from AD Knowledge Portal and GDC Data Portal	GCN and GAT	In the analysis of BRCA, the identified biomarkers demonstrated strong alignment with clinically significant pathways and actionable drug targets, thereby reinforcing the rationale for subtype-specific interventions, including HER2-directed therapies. Furthermore, PPI analysis pinpointed key hub genes—including CCNA2, CDK1, and ESR1—which align with established cancer hallmarks and known drug targets.	[117]
Omicsformer	Datasets from blood and urine samples of high-altitude acclimated populations	Transformer	The model successfully identified critical molecular drivers across diverse pathological conditions. Specifically, it highlighted HEXIM in cardiac hypertrophy, TG and PC metabolites in cardiovascular and liver diseases, and FOXO3 in Alzheimer’s disease (AD) pathogenesis.	[118]
TransFuse	Datasets from the ROSMAP cohorts	GNN	Key discoveries included the APOE-EGR1 interaction and the link between MAPT and phosphorylated tau peptides. Elucidated a neuroinflammatory axis involving APP, CD44, and EGR1, alongside ANGPT2-PIK3R1 connectivity. Validation via eQTL analysis confirmed tissue-specific SNP effects in the frontal cortex, while pathway enrichment highlighted dysregulated VEGF and EPH signaling as fundamental drivers.	[119]
CardiOmicScore	UK Biobank data	multitask deep neural networks	Identification of high-priority CVD-related proteins and metabolites representing promising data-driven molecular pathways associated with cardiovascular risk, leading to the discovery of novel biomarkers and targeted therapeutic candidates to facilitate precision medicine for the primary prevention of CVDs; these findings uncover specific molecular signatures that call for further external validation to solidify their clinical utility in early intervention.	[120]
SLGNN	SynLethDB	GNN	Outperformed five SOTA methods (AUC: 0.9635, AUPR: 0.9710, F1: 0.9089), demonstrating the superiority of encoding semantic information from Knowledge Graphs. Enhanced interpretability by modeling SL-related factors as weighted combinations of KG relations; specifically, it revealed that gene-regulation and covariance relationships (Factor p_3_) were the primary drivers for the interaction between gene_357_ and gene_33124_, offering mechanistic transparency.	[121]
MLEC-iSL	Datasets from CCLE, DepMap, BioGRID, etc.	Multi-layer Encoder (Gene Encoder, GCN and Graph Transformer)	Validation via a CRISPR-Cas9 double-knockout (CDKO) screen demonstrated that MLEC-iSL-guided selection yielded a synthetic lethality confirmation rate of 46.8% (462/987 pairs), marking a substantial improvement over the 7.2% baseline rate (88/1225 pairs) observed in unguided experiments.	[123]
KG-Slomics	Datasets from DepMap, SynLethDB v2.0 and SLKB	Knowledge Graph-based GAT	Achieved superior predictive accuracy and identified the TP53-PDGFRB synthetic lethal interaction. The biological relevance of this interaction was validated through patient survival stratification and drug response analysis.	[124]
MVGCN-iSL	Datasets from CCLE, DepMap and BioGRID	multi-view GCN and DNN	The model was used to conduct experiments on two cancer cell-lines (K562 and Jurkat) individually. For the K562 cell line, 1523 of 100,128 samples (1.5%) are SL gene pairs, whereas only 373 of 74,691 samples (0.5%) in the Jurkat cell line are SL gene pairs.	[125]
SLWise	Datasets from DepMap (22Q2), LINCS L1000, TCGA (cBioPortal), etc.	GNN and Transformer	Identified a cell-specific SL interaction between BCL2L2 and WEE1 in the A375 cell line. Validation revealed that the knockdown of these genes induced abnormalities in key drivers CNOT9 and RHOA. Notably, RHOA was highlighted as a pivotal therapeutic target due to its established role in tumor progression and metastasis.	[126]
GNNRAI	Datasets from AD Knowledge Portal	GNN	Utilizing the integrated Target Risk Score, the framework prioritized APP, APOE, LGMN, and LTF within the top 2% of candidates. These rankings are biologically coherent with established Alzheimer’s pathology: APP and APOE are recognized drivers of Aβ and tau aggregation, while LTF is a known indicator of amyloid burden.	[127]
P-NET	Datasets from The Reactome pathway knowledgebase, etc.	Artificial Neural Networks (ANN)	By assigning differential weights to molecular features based on predictive utility. In the context of CRPC, the model prioritized MDM4 as a critical therapeutic target in CRPC. Experimental validation confirmed that MDM4 inhibitors serve as an effective precision therapy for TP53-wild-type patients, while also successfully corroborating established drivers like AR and PTEN.	[128]
PI4AD	Datasets from NHGRI-EBI GWAS Catalog, STRING database, etc.	ANN	PI4AD successfully recovered clinically validated targets within the top 1% of prioritized genes, assigning high rankings to APP (18th), ESR1 (61st), and PDGFRB (100th), which are targets of drugs in Phase III trials or approved clinical use.	[129]
A deep autoencoder-based computational framework	Datasets from DrugBank database, etc.	AE	Prioritized 187 putative targets, with top-ranked candidates including DLG4 (1st), EGFR (3rd), SYK (4th), and PTK2B (5th). Literature validation confirmed their critical roles in AD pathogenesis: epigenetic editing of DLG4 ameliorates cognitive decline, while EGFR inhibitors prevent memory loss. Additionally, RAC1 and SOCS1 were highlighted as key modulators of neuronal cell death and neuroinflammation, respectively.	[130]
SLMGAE	Datasets from SynLethDB, SynLethDB-BC and GIs	Multi-view Graph Auto-Encoder	Predicted novel SL candidates, identifying 30 literature-supported pairs among the top 1000 predictions. Validated 13 pairs through wet-lab evidence, specifically highlighting KRAS-driven synthetic lethality such as BRCA1-KRAS and KRAS-UNC13B (confirmed by shRNA screening). Furthermore, correctly predicted HDAC9-MYC (validated via drug inhibition) and AKT1-CHEK1 (CRISPR-Cas9), proving the model’s ability to discover biologically actionable targets.	[131]
NETTAG	GWAS Catalog, GTEx	GNN	By integrating multi-modal genomic data (e.g., epigenomics, transcriptomics) with the human interactome, the model prioritized 156 putative risk genes. A key achievement of this study was the identification of MEF2D and CPLX2 as novel therapeutic targets.	[132]
NDSP	Datasets from GDSC dataset, BioStudies, Cell Model Passports and GEO	AE	NDSP demonstrated the capacity to select pathway-relevant gene sites for lung cancer. Key discoveries included the identification of the ERAD pathway from RNA-seq data, which was validated via DisGeNET to be associated with non-small-cell lung cancer. Additionally, the model highlighted HIF-1 survival signaling in CNA data as a significant driver of tumor development, validating the biological relevance of the selected features.	[133]
FI-Net	TCGA	ANN	All driver genes identified by FI-net in 15 of 31 datasets were also detected by other methods. For example, FI-net identified known driver genes, DNMT3A, FLT3, NPM1, IDH1, IDH2, TET2, TP53, RUNX1, CEBPA, NRAS, WT1, U2AF1, and KRAS, in LAML, which were detected by at least 10 other methods and presented in CGC and NCG database.	[134]
GGraphSAGE	Datasets from TCGA, STRING, intOGene, OMIM, DriverDBv3 and NCG	GNN	The model identified multiple novel predicted cancer driver genes (NPCDs), with representative examples including CUL1 and ACTN2, which were supported by cell proliferation association analyses and prior experimental studies.	[135]
deepCDG	Datasets from GDC, NCG(v6.0), COSMIC(v91), DigSEE, KEGG, CPDB, MULTI-NET, STRING and IRefIndex	GCN	Demonstrated superior performance across 15 cancer types using tri-modal omics features (mutation, methylation, expression), achieving the highest AUPRC in 11 types and showing robustness against class imbalance. Identified 148 novel candidate driver genes, of which 86.5% were validated against literature-derived databases (CancerMine and CCGD), confirming their biological relevance to cancer progression.	[136]
DeepCDR	Drug Structure, Mutation, Expression, Methylation	Uniform Graph Convolutional Network (UGCN)+CNN	Developed a gradient-based prioritization strategy (measuring prediction sensitivity to gene expression) to uncover therapeutic targets. Validated by correctly ranking known targets as top drivers: EGFR ranked 1st for Erlotinib (in A3/KAW cells) and 4th for Lapatinib (in BT-474 cells); also identified ABI1 (Rank 2) as a key susceptibility gene for Nilotinib in leukemia lines.	[137]
EMOGI	TCGA	GCN	EMOGI leverages multi-omics pan-cancer data and protein–protein interaction (PPI) networks to identify cancer drivers. By aggregating top-ranked predictions across six PPI networks and filtering out known drivers, the authors identified 165 novel potential cancer genes (NPCGs).	[37]

## Data Availability

No new data were created or analyzed in this study.
